# The impact of exercise training complementary to early intervention in patients with first-episode psychosis: a qualitative sub-study from a randomized controlled feasibility trial

**DOI:** 10.1186/s12888-019-2179-3

**Published:** 2019-06-21

**Authors:** Lene Q. Larsen, Helle Schnor, Britt P. Tersbøl, Bjørn H. Ebdrup, Nikolai B. Nordsborg, Julie Midtgaard

**Affiliations:** 1 Department 9701, The University Hospitals Centre for Health Research, Copenhagen University Hospital, Rigshospitalet, Blegdamsvej 9, DK-2100 Copenhagen Ø, Denmark; 2 Mental Health Centre Glostrup, University of Copenhagen, Nordstjernevej, DK-2600 Glostrup, Denmark; 3 University College Copenhagen, Tagensvej 86, DK-2200 Copenhagen N, Denmark; 4 Global Health, Department of Public Health, University of Copenhagen, Øster Farimagsgade 5, DK-1353 Copenhagen K, Denmark; 5 Center for Neuropsychiatric Schizophrenia Research, CNSR, and Center for Clinical Intervention and Neuropsychiatric Schizophrenia Research, CINS, Mental Health Centre Glostrup, University of Copenhagen, Nordstjernevej, DK-2600 Glostrup, Denmark; 6 Department of Clinical Medicine, University of Copenhagen, Faculty of Health and Medical Sciences, Blegdamsvej 3B, DK-2200 Copenhagen N, Denmark; 7 Department of Nutrition, Exercise and Sports, University of Copenhagen, Nørre Alle 51, DK-2200 Copenhagen N, Denmark

**Keywords:** Schizophrenia, Exercise training, Early intervention, Young adults, Recovery, First-episode psychosis, Thematic analysis

## Abstract

**Background:**

Burgeoning evidence suggests that exercise improves physical and mental health in people with schizophrenia. However, little is known about the feasibility and acceptability of high-intensity training in patients with first-episode psychosis. This qualitative study explored motivation, social interaction and experiences of participants and instructors in relation to an eight-week moderate to high intensity exercise training programme in a clinical trial including patients with first-episode psychosis.

**Methods:**

The study used a combination of method, source and investigator triangulation. Data were collected by means of semi-structured individual interviews with participants at baseline (*n* = 16) and at follow-up (*n* = 9), as well as by means of participant observations during the programme (8 sessions × 1.5 h, 12 h in total) and focus group discussions with participants (*n* = 3) and instructors (*n* = 4), respectively, after the programme. Data were analysed using thematic analysis as described by Braun and Clarke.

**Results:**

Three main themes and ten subthemes emerged during the analysis: 1) *motivation and expectations for enrolment* (subthemes: routines and structure, social obligation, goal setting and self-worth); 2) *new demands and opportunities* (subthemes: practicalities of the training, an understanding exercise setting, and alone and together); and 3) *looking ahead – reflections on impact* (subthemes: restored sleep and circadian rhythm, energy and sense of achievement, changed everyday life, and hope of finding a new path). Findings suggest that the programme was appealing to, and appreciated by, the participants because of its potential to create an equally challenging and caring non-clinical environment.

**Conclusions:**

This study indicates that supervised, group-based, moderate to high intensity exercise training complementary to early intervention in psychosis is acceptable. Specifically, the intervention appeared to provide patients an opportunity to integrate the notion of being a young individual along with being a patient with a psychiatric diagnosis, thus supporting and promoting recovery.

**Trial registration:**

ClinicalTrials.gov identifier: NCT03409393. Registered January 24, 2018.

## Background

The schizophrenia spectrum encompasses psychotic disorders, which are serious mental disorders with typical onset in adolescence and in the early 20s, with few being diagnosed after the age of 40 [1]. Although some patients recover, a substantial proportion will experience persistent functional and cognitive impairments [2]. For these reasons, psychotic disorders are ranked among the most burdensome and costly illnesses worldwide [3–5].

Specialised early intervention in psychosis is described as the most successful recent addition to the treatment of schizophrenia [6]. Early intervention in psychosis in Denmark comprises specialised outpatient multidisciplinary teams called OPUS. OPUS is a well-documented, intensive treatment modality for patients with first-episode psychosis (FEP) including schizophrenia [7]. OPUS is not an acronym; the name comes from the world of music and symbolizes the necessity for different instruments to play together [8]. OPUS include treatment with antipsychotic medication, cognitive-based case management, psychoeducational family involvement, and social skills training [9]. All team members, except the psychiatrist, function as primary team member for a given patient, and the caseload is 10:1 [10].

Despite considerable progress in treatment, people with schizophrenia are 2–2.5 times more likely to die earlier than the general population, with an average life expectancy that is 15–20 years shorter [11, 12]. Cardiovascular diseases constitute a significant contributing factor to this mortality gap, which may be partly attributable to weight gain induced by antipsychotic medication [13]. However, also modifiable lifestyle factors such as smoking, substance abuse and physical inactivity play a central role [13].

It is widely acknowledged that physical activity including exercise training is effective in preventing and managing cardiovascular diseases in non-psychiatric populations [14, 15]. Moreover, a recent, large cross-sectional study documents that exercise is significantly and meaningfully associated with fewer days of poor self-reported mental health in the general population [16]. Furthermore, a recent meta-analysis [17] documented that exercise can improve cognitive functioning among people with schizophrenia, particularly in interventions with higher doses of exercise. Currently, however, OPUS does not exercise training as a standard treatment, and, to our knowledge, only one previous study [18] examined the perceived effects of exercise participation as experienced by people in the early stages of psychosis. Firth and colleagues [18] interviewed nineteen people with FEP who had recently participated in a 10-week moderate-to-vigorous exercise intervention. The authors reported that the participants experienced that their mental health improved [18]. Moreover, few non-randomized studies [19–21] that have demonstrated promising results in relation to supervised exercise in young people with FEP, including prevention of antipsychotic-induced weight gain [19], improved cardiorespiratory fitness [20], and symptomatic, neurocognitive and metabolic outcomes [21].

Functional training programmes characterised by functional movements (i.e. movements which replicate activities of daily living) performed at moderate to high intensity and with constantly varying movements, e.g. CrossFit®, constitute a growing fitness regimen [22]. Functional training programmes are well marketed and have become increasingly popular due to their motivational and challenging nature [22]. This may be related to a great sense of community and enjoyment compatible with that presented in sports practice [23]. However, little is known about the acceptability of functional exercise training as an adjunct to early intervention in FEP.

Against this background, we designed COPUS (i.e. Corpus plus OPUS), a randomised feasibility trial intended to examine the recruitment, retention, acceptability and potential efficacy of a supervised, group-based, functional exercise training programme in patients with FEP. While quantitative data related to feasibility including evaluation of possible changes in cardiorespiratory fitness, body composition and muscle strength are reported in a separate paper, the overall aim of the current qualitative sub-study was to provide an understanding of the complexity of the programme. This includes description of contextual factors of importance in the planning and execution of a subsequent Phase III trial and potential programme implementation.

The specific aims of the current study were four-fold:

- To explore motivational perspectives of patients with FEP in relation to participation in a moderate to high intensity exercise programme.
- To describe social interaction among participants (and instructors) during the programme.
- To explore participants’ positive/negative experiences in relation to the impact of participation.
- To explore the perspectives of the exercise instructors responsible for delivering the programme.

## Method

### Parent study: the COPUS trial

The aim of the overall study, i.e. the COPUS trial, was to investigate whether it was possible to recruit and retain patients with FEP for an eight-week, supervised exercise training programme. The COPUS trial included young adults (18–45 years of age) with a recent International Classification of Diseases, 10th revision (ICD-10) diagnosis of F20-F29 (i.e. schizophrenia, schizotypal and delusional disorder, and other non-organic psychotic disorders) [24]. Moreover, to ensure that patients’ treatment was stabilized and to avoid interference with concurrent clinical trials, patients were required to have been enrolled in OPUS for at least 6 months corresponding to a minimum of 24 consecutive weeks of treatment with antipsychotic medication based on individual clinical needs. Only exclusion criteria included physical contraindications for exercise participation, pregnancy (self-reported), and inability to give informed consent. Study participants (*n* = 25) were recruited by OPUS staff members and randomly assigned to the intervention group (*n* = 13) or a waiting-list control group (*n* = 12). COPUS was approved by the Danish Data Protection Agency (file no.: 2012-58-0004) and the National Committee on Health Research Ethics (H-17018798). The ClinicalTrials.gov identifier is NCT03409393.

#### Intervention and setting

The COPUS trial consisted of 8 weeks of multifaceted moderate to high intensity exercise training inspired by CrossFit®, which was carried out in a commercial fitness centre three kilometres away from the OPUS facility. Two teams comprised of two instructors, who were undergraduate students at the University of Copenhagen Department of Nutrition, Exercise and Sports, supervised the programme. The training sessions comprised of 1 hour three times a week (twice a week in the morning and once a week in the afternoon). Each session began with warm-up exercises and then had one to two games (e.g. a game of tag) followed by the workout of the day, often consisting of high-intensity circuit training, before concluding with stretching exercises. Participants were given the chance to participate free of charge in Copenhagen Warrior®, a six-kilometre race with approximately 30 obstacles, to mark completion of the trial and to give them an end goal.

### Qualitative sub-study

**Fig. 1:**
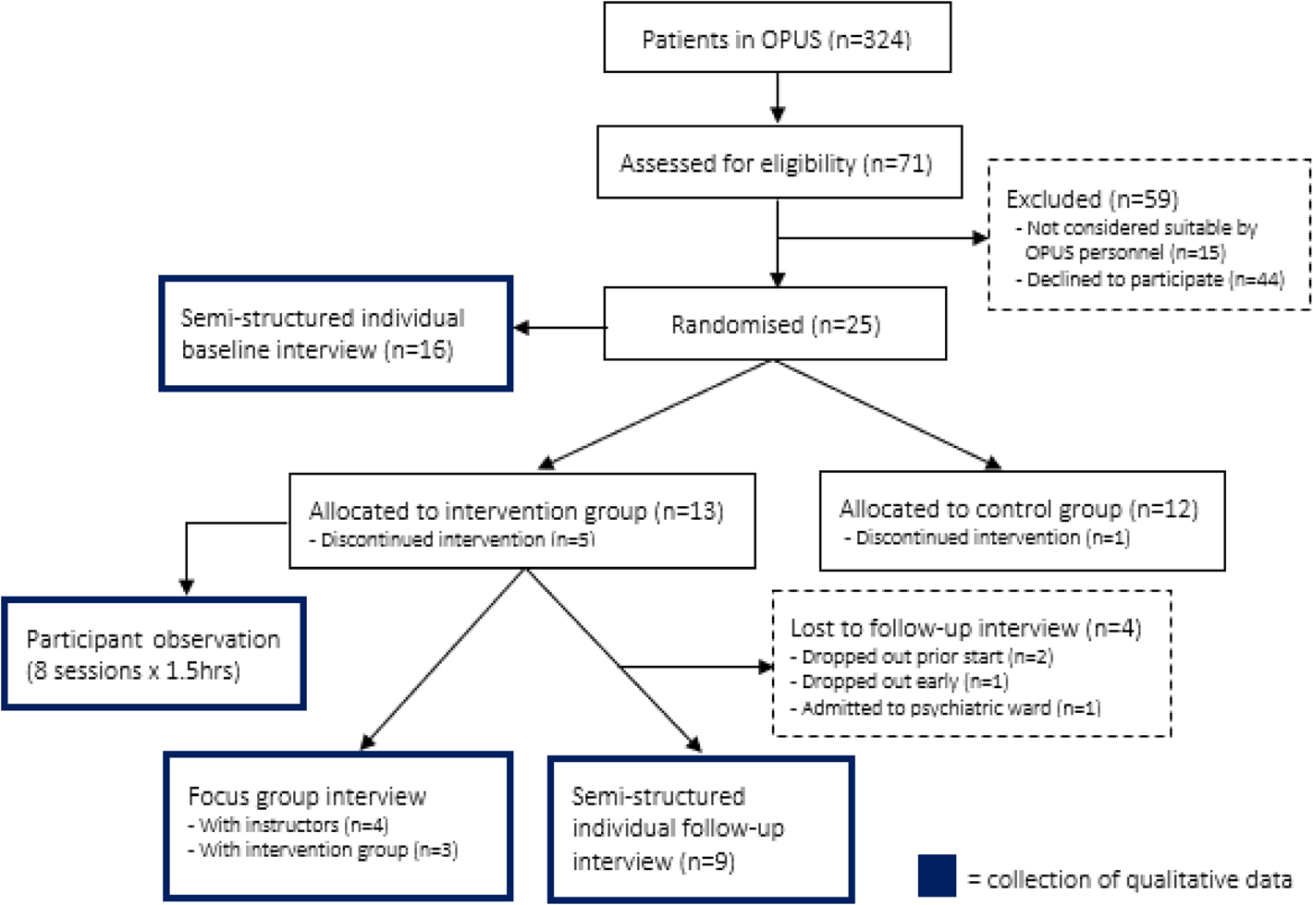
Flowchart of participants during the study and points of qualitative data collection

The current qualitative study used a combination of method, data and investigator triangulation, and was carried out during the COPUS trial to explore and describe the complexity of the intervention including behavioral processes related to delivery and potential impact [25]. Figure 1 provides an overview of the study in relation to the parent study. No separate ethical approval was required for the current qualitative sub-study; however, each participant was informed that they were not required to accept the invitation to take part in interviews, and that potential withdrawal from interviews thus would neither affect their participation in the overall trial nor influence their general OPUS treatment. Furthermore, none of the instructors and/or researchers involved in the current study knew the participants beforehand or were involved in care and treatment of participants.

#### Sampling

Criterion sampling were used to purposefully review and study cases that met predetermined criteria [26], i.e. the COPUS trial inclusion criteria described above. Moreover, participants were sampled conveniently implying that only participants who were available on the days that were scheduled for interviews, were included.

#### Data collection

Data were collected from February 2018 to April 2018 and included semi-structured individual interviews, focus group discussions and participant observations. Table 1 provides an overview of the data collection methods and themes for the interview guides and observation guide, respectively. Except for baseline interviews, all data were collected by the first author, LL, who was not otherwise involved in the parent study including planning and delivery of the intervention.

**Table 1:** Data colletion methods and topics

Specific objective	Data collection method	Topics covered by interview/observation guide	Data source	Time frame
Participants’ motivation and expectations	Individual semi-structured interview (*n* = 16)	(1) motivation for participation, (2) expectations, (3) prior training experience	Study participants	Baseline (prior to randomisation)
Interaction within the intervention (group dynamic, norms)	Participant observation (8 sessions × 1.5 h)	(1) participants entering and exiting the room, (2) information to participants from instructors, (3) informal conversations, (4) non-verbal expressions (i.e. facial expressions and bodily movements), (5) physical surroundings (i.e. room, light, temperature)	Intervention group and instructors	During intervention (week 0–8)
Instructors’ experiences of their role and responsibilities	Focus group discussion (*n* = 4)	(1) expectations and prior coaching experience, (2) experience with the programme; (3) roles and authority, (4) advice for future instructors for patients with FEP	Instructors	After intervention (after 8 weeks)
Participants’ evaluation of the intervention	Focus group discussion (*n* = 3)	(1) advantages and disadvantages, (2) social norms and social cohesion, (3) relation to and role of instructors, (4) suggestions for improvement of the intervention	Intervention group	After intervention (after 8 weeks)
Possible impact and change in subjective wellbeing	Individual semi-structured interview (n = 9)	(1) the programme in general (positive and negative experiences), (2) social aspect of the training, (3) body and mind, (4) impact on symptoms and daily life	Intervention group	After intervention after 8 weeks)

#### Semi-structured individual baseline interviews

In total, 16 participants were interviewed at baseline to explore past exercise experiences, motivation for enrolment in the study and expectations concerning the programme. Baseline interviews were scheduled to take place on four random days over a period of two weeks. This meant that each participant who showed up for baseline testing at the OPUS facility on one of those days was approached by a member of the research team and invited to an interview, which was conducted on-site in a quiet room.

#### Participant observation

Eight participant observations lasting one and a half hours each were conducted throughout the programme to gain first-hand insight into the social interaction, including how the instructors delivered the training and how participants received it. The observations ranged from passive observation with, with the researcher taking a bystander role, to active participation, where the researcher participated on equal terms with the study participants in the training. Participant observation included taking fieldnotes during and immediately after observations.

#### Semi-structured focus group discussion with intervention group

One focus group discussion was conducted after the programme’s last training session. Three participants showed up for the last training session and all were able and willing to stay for an extra hour to take part in the focus group discussion which focused on allowing participants to mutually discuss and reflect upon how they experienced the programme.

#### Semi-structured focus group discussions with instructors

Two focus group discussions were conducted with the instructors, one with each team, i.e. there were two instructors at each interview, at the University of Copenhagen. The specific aim of interviewing the instructors was to gain insight into how they experienced their roles throughout the programme, including responsibilities and their position of authority.

#### Semi-structured individual follow-up interviews with intervention group

Participants allocated to the intervention group were contacted individually and invited to an individual follow-up interview aiming to provide insight into positive and negative experiences concerning participation in the programme. Four participants, three of whom did not complete the intervention, did not respond to the invitation for an individual follow-up interview. Thus, a total of nine interviews were conducted; seven at the OPUS facility and two over the phone.

### Data analysis

All interviews were audiotaped and transcribed verbatim, after which the first author checked the transcriptions against the recordings. Braun and Clarke’s thematic analysis approach was used to jointly analyse the fieldnotes from the participant observations and the interview transcriptions [27]. The analysis was an iterative process that included the following six steps: *1. Familiarisation:* To become immersed in and intimately familiar with the data, we initially individually noted down ideas about possible themes during the transcription (LL only), reading and re-reading of the data.*2. Coding:* Using the software programme NVivo 11, LL systematically coded the data for common, interesting features. *3. Searching for themes:* LL, HS and JM each examined preliminary codes to determine prevalent themes. *4. Reviewing themes:* LL, HS and JM reviewed initial themes together, with LL subsequently reviewing themes in NVivo 11 to check whether they worked in relation to the previously coded extracts and the entire data set.*5. Defining and naming themes:* LL, HS and JM carried out an ongoing collaboration analysis to refine the specifics of each theme and the overall story of the analysis. This involved reorganising and rewriting to achieve coherent links between main themes and subthemes.*6. Writing up:* Themes were rewritten with an analytic narrative to achieve a coherent story.

## Results

Sixteen participants (12 women, 4 men, mean age 25.0 ± 4.5 years) were interviewed at baseline of whom 13 were randomised to the intervention group. Of these, nine participants (6 women, 3 men, mean age 24.3) were interviewed individually after completing the programme, including one participant who had dropped out after the first training session. Table 2 provides an overview of the medical and sociodemographic characteristics of the participants.

**Table 2:** Participant baseline characteristics

Characteristics	Participants interviewed at baseline (*n* = 16)
Age (years); mean *± SD*	25.0 ± 4.5
Gender	
Men, n *(%)*	4 *(25.0)*
Women, n *(%)*	12 *(75.0)*
Body mass index, mean *± SD*	26.1 *± 5.6*
Education level, n *(%)*	
8th, 9th,10th grade	6 *(50.0)*
High school	3* (25.0)*
Vocational education	2 *(16.7)*
Short higher education < 3 years	1 *(8.3)*
Residence, n *(%)*	
Own residence	7 *(58.3)*
Rented room	2 *(16.7)*
With parents or family	3 *(25)*
Diagnosis, n *(%)*	
F20 schizophrenia*	7 *(50.0)*
F21 schizotypal*	6 *(42.9)*
F29 non-organic psychoses*	1 *(7.1)*
Months since starting at OPUS, *mean ± SD*	9.6 *± 4.3*
Months since diagnosis, *mean ± SD*	7.8 *± 3.9*
Activity, n *(%)*	
Almost completely inactive	2 *(15.4)*
Moderate (2–4 h a week)	5 *(38.5)*
Moderate (4 h a week)	4 *(30.8)*
More strenuous (more than 4 h a week)	2.2 *(15.3)*
Smoking, yes/no *(%)*	5/11 *(31.3)*
Cannabis, yes/no *(%)*	1/16 *(6.3)*

*Diagnosis code as defined by the International Classification of Diseases, 10th revision

**Fig. 2:**
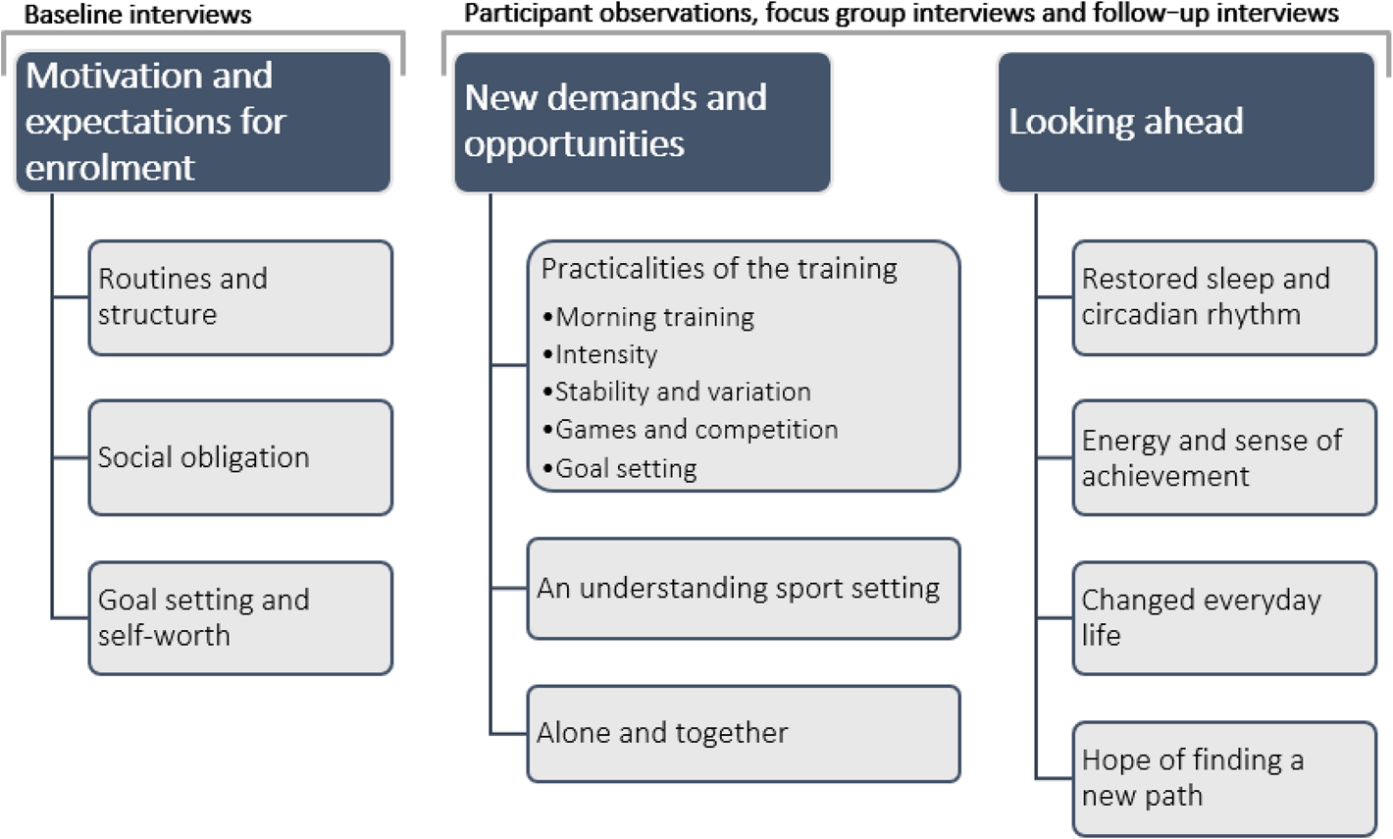
Main themes with subthemes

The analysis resulted in three main themes and ten subthemes, which are described below and illustrated in Fig. 2. In the following, quotes by participants are written in italics and Table 3 provides additional examples of especially illustrative quotes and fieldnotes.

**Table 3:** Illustrative sample quotations and fieldnotes according to main themes

Main theme	Quotations (samples)	Fieldnotes (samples)
Motivation and expectations for enrolment	*I’m ashamed of what has happened after all those years with no training.* (…) *I’ve gained weight and haven’t really been doing anything, also physically. So, I think if or when I complete this, I’ll feel much better about myself. It’ll be kind of a proof that I still can.*	N/A
	Baseline interview, P4 (male, 26)	
	*And then I would also like to train, but I’m not that good at getting out the door, so I want to take the initiative myself. So, if I’m with someone, especially someone I know, then I want to know and get it done.*	
	Baseline interview, P6 (female, 19)	
New demands and opportunities	P5: *Well, it’s kind of a better start to the day; when the training is later, you have to wait a few hours and then you start to think so many thoughts in your head about it and stuff like that.* P8: *Yeah, and then the day is almost gone already.* P1: *Yeah, you haven’t done anything and just vegged all day.* P8: *But in the morning, you come home at a time when you would’ve normally woken up.*	In the changing room, P5 told me it was more intensive than the other training sessions with the male instructors, and that this kind of workout of the day was good because it sparked some competition. As she said: “*You don’t want to be the last one finishing, so it motivates you to do it faster”*.
	Focus group discussion with participants	
	P1: *I’m very pleasantly surprised because I was afraid we would start really slow, so it’s been really good. And I actually think that - I know that it sounds strange - but we haven’t been treated like sick people; sometimes you meet people who talk to you as if you were stupid.* P8: *Or a four-year-old child. (...) Yeah, I don’t think I’ve been treated differently.*	Participant observation, 12 March 2018
		When P3 and P1 entered the changing room, they talked about how tired they felt and that the training had been intensive today. Then Ellen said she was looking very much forward for Copenhagen Warrior® – “like a little child”, to which P3 replied that she was also looking very much forward to it, while P1 nodded in agreement.
	Focus group discussion with participants	
	*I think it’s nice that you don’t have to explain. We’re still very different people and have very different symptoms, but basically, we know that we’re all fighting with the same things and that really helps me. That I can just say ‘it’s just a shitty day today’ and then people don’t ask for an explanation, because ‘well I recognise that, I had that yesterday’ or something like that. Or you can say I’m in a really bad period right now and then they just know what you mean. I think that’s really nice.*	
		Participant observation, 12 March 2018
		One instructor put on a song and everyone was running around the room to the song. When a specific word was sung everyone had to do a push-up. The instructor decided on what kind of running everyone had to do (e.g. backwards or to the side) and also told everyone to drop to the ground when the word was sung. She continued to encourage the participants to keep running and to do push-ups.
	Follow-up individual interview, P1 (female, 35)	
	*You relax more instead of just sitting down and having to talk and stuff like that. Here you’re doing something together.*	
Looking ahead – reflections on impact	Follow-up individual interview, P5 (female, 27)	Participant observation, 14 March 2018
	*I’m tired in the evening. I’ve had a lot of trouble sleeping, but now I’m tired in the evening and I go to bed at a fitting time and get up early. And I also have more energy to do the work I occasionally have.*	After a short break, the workout of the day was introduced and differed compared to other sessions. This time everyone had to do three rounds of the same exercises at the same time with a one-minute break in between. The round comprised 10 push-ups at end of the room and running to the next post in the middle of the room to do 20 lunges per leg before moving on to do 30 mountain climbers and then 40 steps on a step bench at the other end of the room.
	Follow-up individual interview, P4 (male, 26)	
	*I become energised. After training I can conquer the whole world. I get home and start cleaning, vacuuming, doing the dishes and the laundry, and I suddenly have the energy to do all these things. So, training definitely energises me, and after I’ve done all the things I needed to do, I go for a long walk. (…*) *It helps to prove it’s me who’s in control and me who decides, and it’s just something that’s in my head, it’s not real. (…*) *It’s my life, my body and me who’s in charge, it’s not my disease that decides over me, it’s me who decides over my illness. So, after I’ve started training I can feel I’m more in control of myself*.	
		The male participants, P2 and P4, did the exercises very meticulously at their own pace and fell behind quite quickly. The three women accompanied one another, with P1 and P3 counting down together, encouraging each other and high-fiving each other after each exercise post, whereas P5 did the exercises and counting down by herself.
	Follow-up individual interview, P3 (female, 27)	
		Participant observation, 12 March 2018
		N/A

### Main theme 1: motivation and expectations for enrolment

#### Routines and structure

The participants’ motivation for enrolment revolved around a wish to gain more structure and routines in everyday life. Several participants expressed a wish to break out of a negative circle of doing nothing or very little. They described feeling that their days were alike, with little progression, and one participant explained how being on sick leave had resulted in her rarely leaving the house. Even though she liked being physically active, she acknowledged that, “*I wouldn’t be able do it by myself*”.

#### Social obligation

For some participants it was important that they knew each other beforehand (i.e. from OPUS). They anticipated that the intervention would create a sense of safety, commitment and social obligation and that they could *“help raise each other up a little”*. Some described how they would probably stop coming if they did not know the others, as it would make them feel lonely. Moreover, some hoped that exercising with people who they knew suffered from the same illness would make it easier to show up for sessions as it would not require explaining if they were having a bad day.

#### Goal setting and self-worth

Several considered the programme to be a goal to accomplish, which they hoped would make them feel better and lose weight. Although most of them appreciated that the programme offered exercise, none specifically mentioned being attracted by the programme being promoted as CrossFit® inspired training. Participants talked more about the programme being an opportunity to challenge themselves and improve their self-worth, e.g. one participant who had previously boxed at a professional level and had gained weight after stopping when becoming ill, expressed: *“I hope this programme will make me start believing in myself again”.* Some participants also said that by doing something active that they knew was good for themselves and their bodies, they hoped it would make them *“feel more human”*. One participant also explained that by challenging her body and feeling sore, she would *“feel more alive”*.

### Main theme 2: new demands and opportunities

#### Practicalities of the training

Several practicalities of the programme were experienced as important for participant adherence to the programme. First, the majority explained that they favoured the morning sessions because they “*did not have time to overthink or make excuses for not attending”*. One participant explained that attending morning sessions meant that there were fewer people on public transportation and at the fitness centre, which, in his opinion, could otherwise deter some from coming due to social anxiety.

Second, participants with prior training experience explained that they liked very intensive training and appreciated being physically challenged. One participant emphasised that the programme was especially good for her as it was so intensive that she did *“not have the energy for anything other than focusing on breathing”*. A few stated that they would have preferred more low-intensity activities before initiating the intervention. For one participant in particular, the programme was experienced as *“too much”* and resulted in her dropping out after the first training session.

Third, several participants said that the training was good because there were several different components and variation, which they thought made it more fun. Concurrently, some said they appreciated how structured the training was. Knowing what to expect made them feel safe and allowed them to *“put the various features of the sessions into boxes”* and prevent building up anxiety. Similarly, the instructors were highly aware of the important role they had in creating what one instructor called *“a safe space”*, in which the participants felt comfortable about participating.

Also, several participants described how the games and competition helped to push them to do more. This also transpired in the observations, where some participants would at times exclaim, to their own surprise, how breathless they were after playing a game. Furthermore, through observations, it became evident that the games often worked as an ‘icebreaker’. Before the games the participants were often quieter and did not smile as much as they did after the games, at which point they would often laugh, grin and joke more among themselves.

Lastly, most of the participants said that the obstacle course race, Copenhagen Warrior®, was *“a major personal goal to accomplish”*. Also, some stated that having the race as a joint goal to achieve together was *“a great thing to work towards as a team”*. This also became evident during observations in the changing room, where the participants sometimes talked excitedly together about their expectations for the race. Yet, some had little interest in Copenhagen Warrior®, with one person forgetting about it entirely and another not feeling fit enough to complete it.

#### An understanding exercise setting

Most of the participants appreciated the role of the instructors, who they felt treated them as “*normal*”. According to the participants, it was important that the instructors challenged them to do more and, in general, that they did not treat them differently, which was also reflected upon in the focus group discussion with the participants. Some also said that they appreciated that the instructors participated in the sessions because it often set the pace of the training and made them go *“all in”*.

The instructors’ experiences of their roles varied. In general, two instructors reflected more on the actual training and saw their main role as being supportive and guiding, whereas the two others reflected more on whether the individual was managing.

Some of the participants said they liked the fact that the programme took place in a fitness centre outside of OPUS, allowing them to *“interact with the real world”*, while still being in a safe and understanding environment. All four instructors explained that maintaining a balance between pushing and being understanding had been difficult, but also an important area of focus in the training. As one instructor explained, *“They don’t need to face their illness; it has to be a free space where they don’t have to think about that”*.

#### Alone and together

Observations showed that while some participants chose to do the exercises alone, others chose to do them together, which, according to the participants, enabled them to oscillate between socialising and not engaging, depending on what they felt like. Additionally, some explained that partaking in the programme provided a topic for conversation, making it easier to be social in other contexts, e.g. with family and friends.

Some also said that they normally found it difficult to be social but experienced that the programme provided *“an easier way of socialising”* because they were active and doing something together. Some of the participants, however, said that they were not interested in the social aspect of the training, while others said they did not have the capacity to socialise while training.

Some participants also explained that being physically active together removed a “*façade*” they would normally have had, making it more manageable to attend, even on bad days. One participant said that she did not feel she had to live up to a certain ideal, e.g. wear makeup and nice clothes, which she would normally use in case of meetings and activities with other people; she expressed that in the context of COPUS *“you can show up just as you are and don’t need to pretend that you’re having a good day”.*

### Main theme 3: looking ahead – reflections on impact

#### Restored sleep and circadian rhythm

When asked specifically, a majority experienced that their sleep had improved. Some experienced that their excessive thinking had been markedly reduced or even disappeared, helping them to fall asleep earlier and regain their circadian rhythm. One participant explained how he previously rarely felt tired, or *“felt ashamed”* about sleeping, as he did not feel he deserved it. However, by being physically active and getting to know his body in the context of the programme, he now found it easier to accept the need for sleep to recover.

#### Energy and sense of achievement

Most of the participants said that completing a training session led to an immediate boost related to a sense of achievement, giving them the energy to do more. However, two participants did not experience these benefits, one of whom said that she had felt “*more down after a training session*”, while another stated: “*it* [the training] *drained me of the little energy I had”*. The programme had become more of a burden to these two participants, who explained that failing to attend a session resulted in a guilty conscience.

#### Changed everyday life

Several of the participants perceived the training as a practical tool, which helped them take more control over their symptoms, including the addition of more structure and content in their everyday life. One participant explained how going to training helped her turn bad days into better days, improving her mood markedly, causing her to feel that she could *“manage the whole world again”* and not let the illness dictate over her. This experience was echoed by other participants, who explained that managing to attend training had helped them overcome bad periods, *“When I got into it* [the training] *again, I also came out of the other* [a bad period]”.

#### Hope of finding a new path

Several participants stated that participating in the programme had made them feel stronger and more content in daily life, which had contributed to a greater sense of meaning and purpose. Succeeding in attending training several times a week, not only gave them an immediate boost but also served as a way of proving to themselves how much they were capable of. One participant explained that he had acquired *“a taste for competing with myself”*, including healthier eating, smoking cessation and getting fitter. Also, a vast majority of the participants expressed a wish to continue to exercise at the commercial fitness centre with other people after finishing the programme.

## Discussion

The overall aim of this study was to provide an understanding of the complexity and contextual factors related to an eight-week moderate to high intensity exercise training programme in patients with FEP by exploring social interaction and perspectives of participants and exercise instructors responsible for delivery of the programme. Overall, the findings suggest that the programme was appealing to, and appreciated by, the participants because of its potential to create an equally challenging and caring environment.

**Fig. 3:**
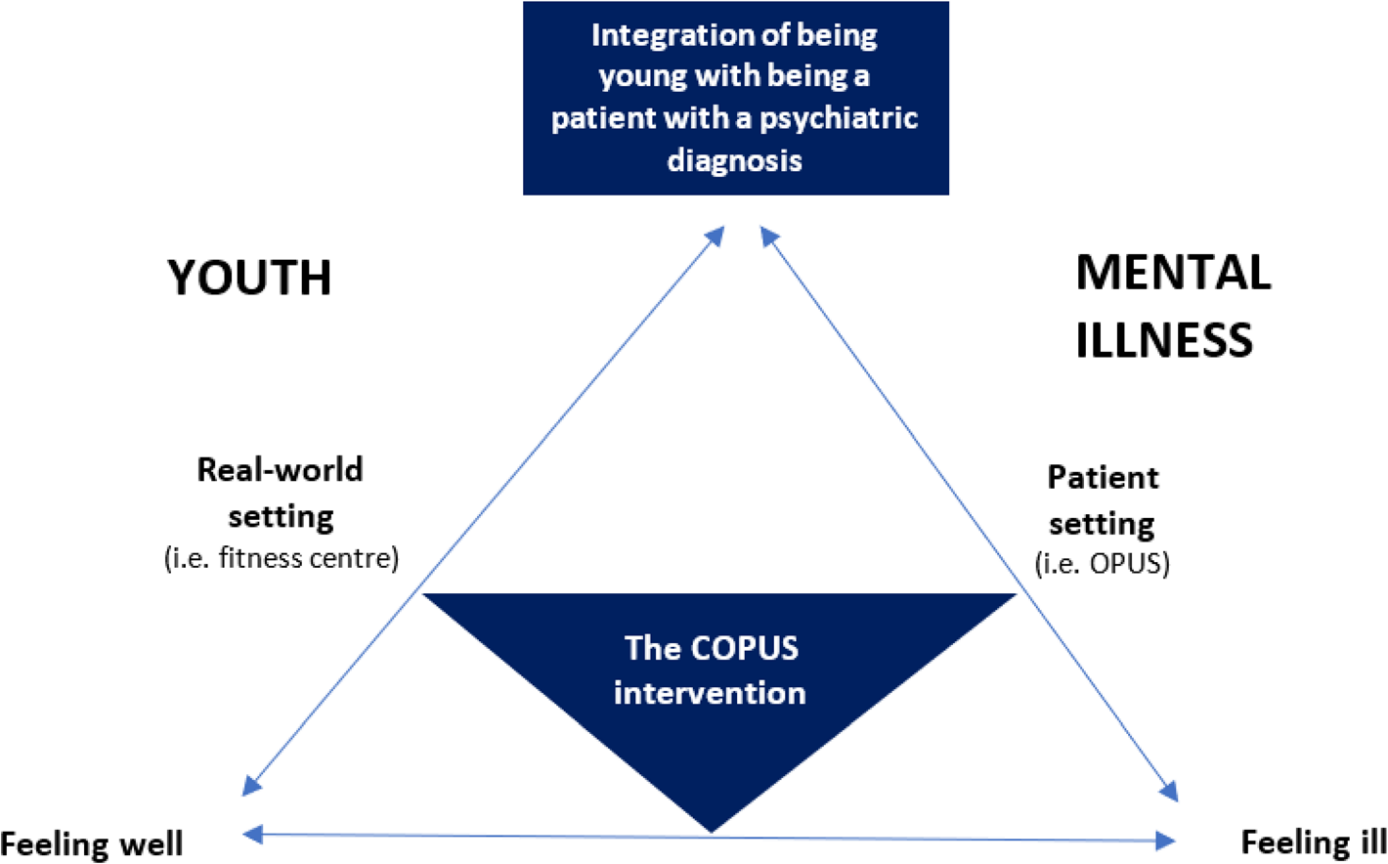
Theoretical model of the potential meaningfulness of the COPUS programme

The fact that the programme was delivered in a non-clinical setting (i.e. a commercial fitness centre), supported the participants in feeling like normal young adults in a real-world setting (i.e. a conventional exercise setting) under the supervision of non-health professionals. The participants’ need to feel safe and to receive care was supported by the fact that the programme was designed, promoted and delivered in collaboration with OPUS staff and that it was exclusively for patients with FEP. Figure 3 provides an illustration of an interpretation of the current study’s main finding, i.e. the programme’s ability to embrace a youth and a mental illness perspective simultaneously.

It is widely acknowledged that young adulthood constitutes a core period for identity development and is often a challenging time [28]. Thus, providing the individuals with FEP the opportunity to better navigate between being a patient with a mental illness and being a young adult could have therapeutic potential, including supporting recovery and preventing social isolation [8].

Specialised early intervention treatment for patients with FEP provides a necessary and imperative focus on schizophrenia, which assist in helping young adults to understand and navigate what it means to be mentally ill [8]. Focusing too greatly on, and being constantly reminded of, being mentally ill may be an unfavourable approach for recovery as doing so may decrease the individual’s feeling of connectedness to peers, potentially disturbing the (re) building of identity, risking that schizophrenia becomes the main identity of the young adult, thereby worsening its severity and chronicity [29].

The findings indicate that exercise training delivered in a non-clinical setting may represent an opportunity for patients with FEP to explore and experience the aspect of being a young adult, with minimal focus on mental illness. The programme offered participants a flexible space that made it possible to integrate being mentally ill and in need of treatment and care as a patient, with being a young adult in need of social inclusion and challenging activities. Because current, specialised early intervention treatments for patients with FEP primarily take place in a patient setting, this programme may provide a novel addition to existing treatment regimens.

To our knowledge, the current study is the first to explore moderate to high-intensity exercise targeting patients with FEP, delivered in a commercial fitness centre *outside* the patient setting. A systematic review and meta-ethnographic synthesis exploring the experiences of people with schizophrenia and exercise, concluded that exercise interventions should be patient-centred to help protect and reassure individuals and to aid adherence [30]. While this is important, our findings suggest that emphasis on experiences of being a normal young adult may be especially important in relation to FEP.

A notable finding of the current study was the contribution of the instructors in creating a caring yet challenging environment. We believe that several factors are important to consider in this regard. First, the instructors supervising the current programme were within the same age-range as the participants. Moreover, they represented an exercise community (i.e. students at the University of Copenhagen Department of Exercise and Nutrition), rather than that of health professionals. This implied that the instructors were unfamiliar with the treatment of mental illness, possibly enabling them to challenge the participants like they would have challenged any other person without mental illness. It is widely acknowledged in the mental health literature that exercise and lifestyle interventions supervised by professionals with relevant training, including physical educators, physiotherapists and exercise physiologists is associated with greater improvements in comparison to unsupervised interventions and/or intervention supervised by other health professionals [31, 32]. Furthermore, previous studies have found that exercise instructors can play an important role in creating a feeling among participants of partaking in a normalised activity, helping to reverse the negative stigma of mental illness [33]. A qualitative review of mental health and physical activity including exercise (in participants with severe and enduring mental health difficulties) also found that exercise instructors played an important role, and were described by participants as key to providing a sense of safety and support [34]. This indicates that non-health professional instructors of exercise programmes in patients with FEP, can play an important role in providing a potentially anti-stigmatising and challenging, yet caring, environment. However, more research is needed to understand the potential benefits related to the involvement of young adults without mental illness as exercise instructors in treatment of FEP.

Another interesting finding was that the participants appreciated that they did not feel like they were being forced to be social because the purpose of the programme was exercise training. Participants explained that the programme provided and promoted easy social interaction, which is consistent with previous studies, which found that exercising provided a safe and meaningful opportunity for interaction, where mental illness did not matter [34]. Participants also explained that participation in the programme provided a conversation topic in other social settings, i.e. at OPUS or with family and friends, making it easier to be social in other contexts as well. Additionally, the participants appreciated the circuit training component, as it allowed them to do the exercises either alone or together. While other types of group-based exercise programmes may be especially motivating, our findings indicate that providing the individual with the flexibility to oscillate between socialising or not may be especially relevant in patients with FEP. This is confirmed by other studies, which indicate that social interaction is particularly difficult for people with schizophrenia and often results in withdrawal from social interactions leading to self-isolation [8], which means it may constitute a barrier for exercise participation. On the other hand, offering people with mental illness the opportunity to socialise with peers on their own premises is a central part of recovery [29, 35]. A study examining the experience of exercising among men with serious mental illness, found that one of the main motivations for participating in a popular sport was to create and talk about an achievement that was shared *by*, and could potentially be shared *with*, many others in society [36]. In the current study, this specific experience appeared to be shared by both women and men, thus not necessarily gender specific. This indicates that satisfaction of basic psychological needs (including autonomy, competence and relatedness), as suggested by health promotion models of self-determination theory, may explain motivational processes behind exercise behaviour in FEP in the same way as in the general population [32, 37]. However, more research is warranted in relation to how participation in peer-based exercise among people with serious mental illness can be applied to increase confidence in socialising in other life contexts. Furthermore, long-term studies are warranted to explore potential post-intervention adherence, including the potential implications of exercise for participants’ experience of recovery, i.e. how exercising may be used as a means to improve recovery in association with mental health services [33].

Although the current study does not provide any evidence in relation to the potential efficacy of exercise as complementary to treatment of FEP, it is interesting that the participants described the programme as providing a distraction from symptoms both during and after exercising. Specifically, the structure of the individual sessions, including clear plans and instructions in relation to the day’s workout, appeared to have supported the participants’ feeling of being able to structure their thoughts in way that made prevent anxiety from building up. This is in accordance with a comparable qualitative study targeting people with FEP which found that participating in a 10-week individualised exercise training programme improved participants’ mental health and confidence and gave them a sense of achievement [18]. A qualitative review of mental health and physical activity found that many mental health service users experienced that exercising helped subdue voices/delusions and redirect attention away from their symptoms [34].

### Methodological considerations

This study possesses a variety of strengths and limitations which should be taken into consideration. The current intervention involved social and behavioral processes that are difficult to explore and capture using quantitative methods alone. However, while the qualitative design of the current study provides a broad understanding, it limits specific inferences, i.e. it does not allow causal linkages between participants’ subjective wellbeing and programme adherence.

The triangulation of methods, sources and researchers contributed to ensuring the study’s credibility and confirmability. Specifically, the involvement of researchers representing various professional and scientific backgrounds (i.e. psychology, nursing, psychiatry, sports science) reduced the risk of investigator bias. Furthermore, method, source and investigator triangulation made it possible to ensure rich descriptions of participant behaviour and experiences in relation to the programme.

Dependability was achieved by using overlapping methods (e.g. interviews and observations) and by describing the purpose and focus. Specifically, results from the different data methods supported each other, and the participants generally confirmed each other’s statements in the focus group discussion and in the individual, semi-structured follow-up interviews, indicating high dependability and confirmability. Another strength of the study was the level of credibility achieved through frequent debriefing sessions and peer scrutiny executed by the first author and JM and HS, making it possible to test ideas and interpretations, as well as to reduce the effect of investigator bias [38]. However, a limitation was that because the first author is female, she was not allowed to enter the male locker room to do observations, which may limit transferability.

We randomly selected participants for baseline interviews to support credibility but, unfortunately, only conducted a single interview with a participant who did not complete the intervention. More drop-out interviews and interviews with patients who did not show interest in the study and/or in being referred could have increased our understanding of potential recruitment and adherence barriers, as well as served to increase transferability. To explore possible long-term effects or the lack thereof, long-term follow-up studies with the participants are warranted. Also, it cannot be ruled out that only participants who were especially motivated and/or resourceful were included, and that collaboration with OPUS staff, who were asked to pre-screen participants, mean that some participants were wrongfully excluded. More research is warranted to understand exercise preferences, and potential individual and structural barriers to supervised exercise in patients with FEP.

### Clinical implications

This study provides a complex picture of the possible strengths and weaknesses involved in introducing moderate to high intensity exercise training as an adjunct to early intervention in treatment of patients with FEP, highlighting several important factors to consider for future practice. For example, the current study indicates that training in the morning compared to the afternoon may be preferable to avoid overthinking and to prevent some anxiety among participants, and that some participants may benefit from supplementary low-intensity, relaxation or stretching exercises. Moreover, the findings suggest that offering participants a choice of doing exercises alone or together in pairs may support adherence. Lastly, having a final goal to accomplish individually but still as part of a team and having the programme delivered in a non-clinical environment by non-health professionals may further help to create a positive, non-stigmatising atmosphere.

## Conclusion

Supervised, group-based, moderate to high intensity exercise training delivered by non-health professionals in a non-clinical setting has the potential to provide a normal, non-stigmatising youth environment, potentially supporting physical activity adherence and recovery in patients with first-episode psychosis.

## Data Availability

Due to privacy protection issues and according to Danish legislation, the datasets supporting the conclusions of this article are available only to the researchers involved in the project. However, after the principal researcher (JM) of the COPUS project consults with the review board, data may be available upon reasonable request.

## Abbreviation

FEP: First-episode psychosis
